# A New Case of Herlyn–Werner–Wunderlich Syndrome: Uterine Didelphys with Unilateral Cervical Dysgenesis, Vaginal Agenesis, Cervical Distal Ureteral Remnant Fistula, Ureterocele, and Renal Agenesis in a Patient with Contralateral Multicystic Dysplastic Kidney

**DOI:** 10.3390/diagnostics12010083

**Published:** 2021-12-30

**Authors:** Jin-Hee Yu, Sa-Ra Lee, Heayeon Choi, Kun-Suk Kim, Byung-Moon Kang

**Affiliations:** 1 Department of Obstetrics and Gynecology, Seoul Asan Medical Center, University of Ulsan College of Medicine, 88, Olympic-ro 43-gil, Songpa-gu, Seoul 05505, Korea; ddalki16@gmail.com (J.-H.Y.); philgirl@naver.com (H.C.); bmkang@amc.seoul.kr (B.-M.K.); 2 Department of Urology, Seoul Asan Medical Center, University of Ulsan College of Medicine, 88, Olympic-ro 43-gil, Songpa-gu, Seoul 05505, Korea; kskim2@amc.seoul.kr

**Keywords:** biopsy, cervical dysgenesis, dysmenorrhea, multicystic dysplastic kidney, uterine didelphys

## Abstract

The aim of this study was to present a new case of congenital Herlyn–Werner–Wunderlich syndrome, a rare anomaly of the female reproductive tract, and review the related literature. A 12-year-old girl presented with severe dysmenorrhea since menarche and magnetic resonance imaging showing a bicornuate uterus, double cervix, right hematometra, and hematosalpinx with ipsilateral renal agenesis, accompanied by a remnant distal ureter with hydroureter. A diagnostic cystoscopy and a reduced-port robot-assisted laparoscopy with chromopertubation were performed in order to identify the anomaly. Uterine didelphys and right cervical dysgenesis with ipsilateral vaginal agenesis, cervical distal ureteral remnant fistula, ureterocele, and renal agenesis were diagnosed on the basis of histopathologic findings, and she subsequently underwent a robotic unilateral right total hysterectomy with salpingectomy. This case report reinforces the importance of the intraoperative biopsy for an accurate diagnosis, despite magnetic resonance imaging being considered the gold-standard diagnostic tool.

## 1. Introduction

Herlyn–Werner–Wunderlich (HWW) syndrome is characterized by the triad of uterine didelphys, obstructed hemivagina, and ipsilateral renal agenesis [1,2,3]. However, HWW syndrome can present with a bicornuate uterus. We present a new category of HWW syndrome through a case of uterine didelphys combined with biopsy-proven unilateral cervical dysgenesis, vaginal agenesis, cervical distal ureteral remnant fistula, ureterocele, and renal agenesis in a girl with a contralateral multicystic dysplastic kidney (MCDK) and a history of severe dysmenorrhea since menarche.

The aim of this study was to present a new case of congenital HWW syndrome, a rare anomaly of the female reproductive tract, and review the related literature.

## 2. Case

A 12-year-old girl was referred to our hospital because of abnormal findings on abdominopelvic computed tomography that suggested a transverse vaginal septum or right-sided vaginal obstruction. The patient had a 7-month history of severe dysmenorrhea and prolonged menstrual bleeding since menarche. Magnetic resonance imaging (MRI) showed a bicornuate uterus, double cervix, right hematometra, and hematosalpinx with ipsilateral renal agenesis, accompanied by a remnant distal ureter with hydroureter. MCDK was also noted, despite the patient’s renal function being within normal limits as determined by the blood urea nitrogen (BUN) level of 17 mg/dL and the creatinine level of 0.47 mg/dL.

**Figure 1:**
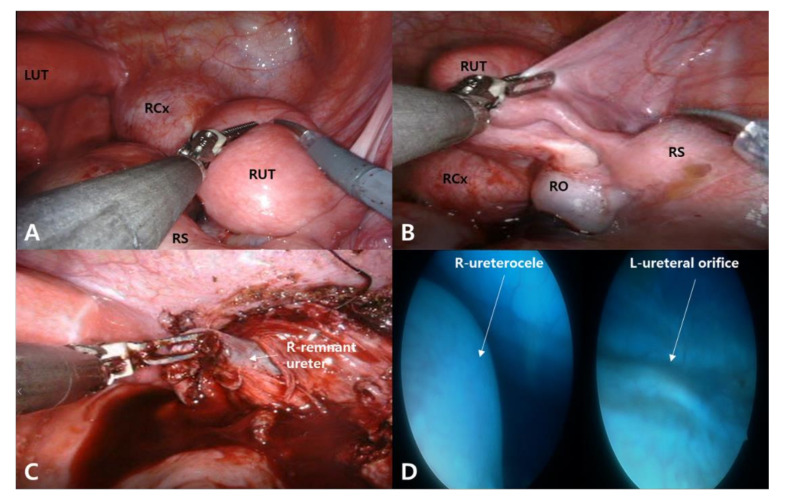
Robot-assisted laparoscopy revealed uterine didelphys. (**A**) A right cervix (RCx) was filled with hematoma and the cervix was obstructed. Ipsilateral hematometra in right uterus (RUT) with hematosalpinx in right salpinx (RS) and normal hemiuterus of left uterus (LUT) were noted. (**B**) Several endometriotic spots on right tuboovarian surface with pelvic adhesions were noted. (**C**) A dilated tubular structure of 15 mm, filled with old blood was noted on the right side of the hysterectomy site. (**D**) Cystoscopy after intravenous injection of indigo carmine dye revealed a right ureterocele and normal left ureteral orifice.

Gross examination under general anesthesia revealed normal external genitalia, including the labia, clitoris, urethra, and a normal cervix and vagina. Hysteroscopy revealed a single uterine cavity with a single left tubal ostium. A 2.5 cm intraumbilical incision was made for the insertion of a multichannel single port for the camera and one robotic arm. One 8 mm incision was made in the right upper quadrant for the insertion of another robotic arm. A reduced-port robot-assisted laparoscopy using the da Vinci Xi system (Intuitive Surgical, Sunnyvale, CA, USA) was performed. “Reduced port” refers to the multiport robotic surgery with a total of two skin incisions, and the patency of the left fallopian tube was confirmed with chromopertubation. There were multiple old blood-colored tinged spots in the peritoneal cavity and uterine didelphys, as well as a right hematocervix with vaginal agenesis, ipsilateral hematometra, hematosalpinx, and several endometriotic spots with pelvic adhesions on the right ovarian surface were noted (Figure 1A–C). The left ovary, hemiuterus, and salpinx showed no gross abnormalities or fluid accumulation. A unilateral right total hysterectomy with salpingectomy was then performed. At the end of the surgery, a cystoscopy was performed after the intravenous injection of indigo carmine dye and it revealed a right ureterocele and a normal left ureteral orifice.

**Figure 2:**
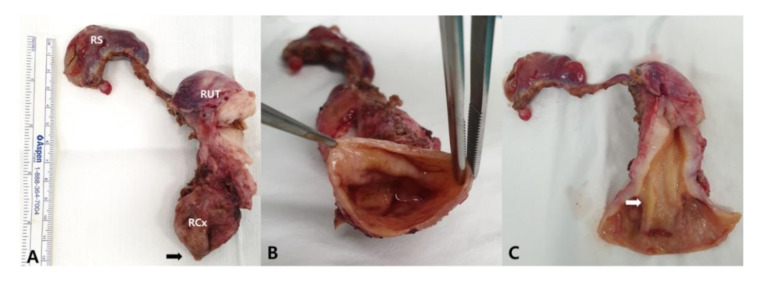
The retrieved uterus and biopsies were performed at two sites (arrows). The biopsied tissues were histopathologically confirmed as the exocervix ((**A**), arrow) and endocervix ((**C**), arrow), respectively. (**B**), Open the hematocervix revealed the dilated cavity. RS, right salpinx; RUT, right hemiuterus; RCx, right cervix.

The retrieved uterus was removed within a bag through the umbilical incision and a longitudinal incision with a surgical blade was made on the specimen to open and expose the uterine cavity. An intraoperative biopsy was performed at both the most caudal end (Figure 2A, arrow) and the most cranial end of the hematosalpinx (Figure 2C, arrow). The squamous epithelium, similar to the exocervix and columnar epithelium and suggesting endocervical tissue, was revealed and confirmed as endocervix and exocervix (Figure 2). The final pathologic diagnosis of the specimen reported uterine corpus and uterine cervical tissue with right hydrosalpinx without a tumor.

**Figure 3:**
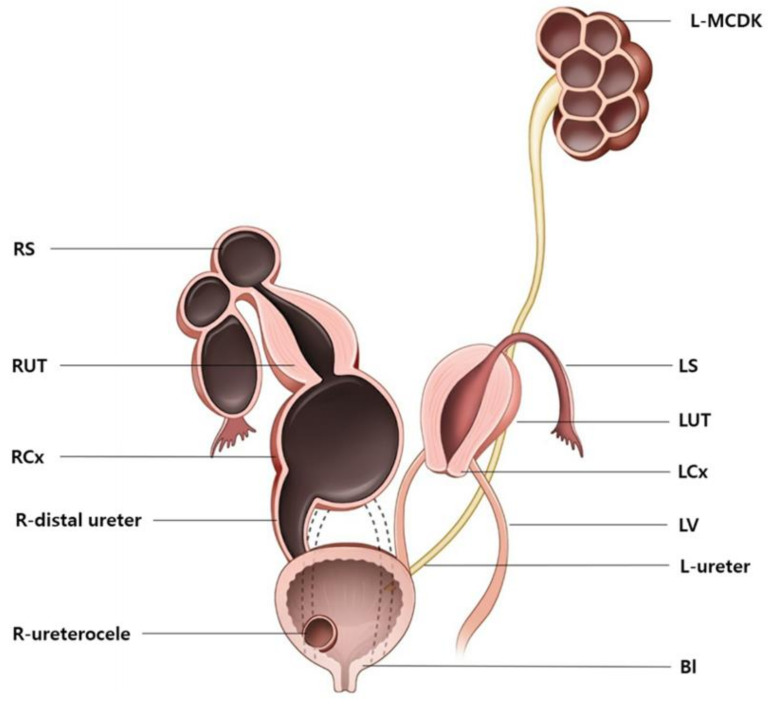
Illustration of this case of a rare category of Herlyne–Wernere–Wunderlich syndrome of a uterine didelphys combined with unilateral cervical dysgenesis, vaginal agenesis, cervico-ureteral fistula, ureterocele, and renal agenesis, combined with a contralateral multicystic dysplastic kidney. RS, right salpinx; RUT, right hemiuterus; RCx, right cervix; R-distal ureter, right distal ureter; R- ureterocele, right ureterocele; L-MCDK, left multicystic dysplastic kidney; LS, left salpinx; LUT, left hemiuterus; LCx, left cervix; LV, left vagina; L-ureter, left ureter; Bl, bladder.

A dilated, blood-filled, 15 mm tubular structure was noted on the right side of the hysterectomy site, which was confirmed via biopsy as the remnant distal ureter, lined with urothelium, which was fistulated to the hematocervix. A cystoscopy revealed a right ureterocele and a normal left ureteral orifice (Figure 1D). The postoperative in-hospital course was uneventful, and the patient was discharged on the second postoperative day. At the 1- and 3-month postoperative follow-up visits, the patient reported normal menstrual periods with only mild dysmenorrhea. At 6 months post operation, renal function was normal as determined by a BUN level of 6 mg/dL and a creatinine level of 0.34 mg/dL. A technetium-99m dimercaptosuccinic acid (Tc-99m DMSA) renal scan also revealed normal renal function with an uptake of 100% for the left kidney, 10 cm in size. To the best of our knowledge, this is the first reported case of a rare category of HWW syndrome involving uterine didelphys combined with unilateral cervical dysgenesis, vaginal agenesis, cervico-ureteral fistula, ureterocele, and renal agenesis in a patient with contralateral MCDK (Figure 3).

## 3. Discussion

HWW syndrome is a rare genito-urinary anomaly that is mostly reported as uterine didelphys, blind hemivagina, and ipsilateral renal agenesis [3,4]; however, its clinical presentations are complex and diverse, including different combinations of uterine anomalies, such as unilateral cervicovaginal obstruction and ipsilateral renal anomalies, including paravaginal cystic structures and/or ureteral remnants [3,4]. Therefore, the absence of uterine didelphys does not rule out HWW syndrome. The syndrome can also be communicant (75%) or noncommunicant (25%) [4]. Symptoms such as dysmenorrhea, dyspareunia, spotting, pelvic mass, and acute abdomen may be observed depending on the degree of obstruction, as seen in other obstructive Mullerian anomalies. As retrograde blood flow might cause endometriosis, hematosalpinx, and pelvic inflammatory disease, most cases of HWW syndrome require surgical treatment.

Ipsilateral renal agenesis is the most frequently reported renal anomaly in patients with HWW syndrome; however, its incidence greatly varies across different age groups. Prepubertal patients mainly show ipsilateral MCDK, whereas adolescent or adult patients show renal agenesis [2,5]. Therefore, MCDK has been proposed to be the precursor of ipsilateral renal agenesis in HWW syndrome [6]. This is the first reported case of HWW syndrome with ipsilateral renal agenesis and contralateral MCDK in an adolescent. Ipsilateral ureteral remnants are usually detected with ectopic ureteral insertion into either an obstructed vagina [7,8,9] or an obstructed cervix, albeit rarely [4]. Among 40 patients with HWW syndrome in a study conducted by Zhang et al., seven showed a dilated ectopic ureter on MRI, and one showed an ipsilateral ureterocele without an ectopic insertion [4].

Unilateral obstruction in HWW syndrome usually involves an oblique vaginal septum, whereas cervical obstruction is rarely observed [4,5,6,9]. Conservative surgical management, such as utero-vaginal or utero-vestibular anastomosis, can be performed for cervical dysgenesis [10]; however, in cases such as ours, with a contralateral normal hemiuterus with a patent and normal fallopian tube, cervix, and vagina, a hysterectomy is considered suitable. In the study by Zhang et al., a hysteroscopic cervicoplasty was performed in seven out of ten surgically managed cervical obstruction cases, whereas a hysterectomy was performed in one case with uterine didelphys, similar to our case [4].

MRI is considered the gold standard in diagnosing congenital or obstructive Mullerian anomalies, such as HWW syndrome, with three-dimensional ultrasonography as a good alternative [11,12,13]. MRI usually shows the exact obstruction sites, providing detailed information for surgical strategy planning [5]. However, considering the results of our previous study, an intraoperative biopsy at the caudal leading edge is recommended for obstructive genital anomalies, particularly for presumptive vaginal or cervical dysgenesis [14].

Our case represents a new category of HWW syndrome: uterine didelphys combined with biopsy-proven unilateral cervical dysgenesis, vaginal agenesis, cervical distal ureteral remnant fistula, ureterocele, and renal agenesis, as seen in a patient with contralateral MCDK.

## 4. Conclusions

As far as we know, this is the first report on a new case of HWW syndrome: uterine didelphys with unilateral cervical dysgenesis, vaginal agenesis, cervical distal ureteral remnant fistula, ureterocele, and renal agenesis in a patient with contralateral multicystic dysplastic kidney. This case report reinforces the importance of the intraoperative biopsy for an accurate diagnosis, despite magnetic resonance imaging being considered the gold-standard diagnostic tool.

## Data Availability

The data used to support the findings of this study were supplied by Sa Ra Lee under license, and requests for access to these data should be made to Sa-Ra Lee, leesr@amc.seoul.kr.
